# Identification of macrophage correlated biomarkers to predict the prognosis in patients with intrahepatic cholangiocarcinoma

**DOI:** 10.3389/fonc.2022.967982

**Published:** 2022-09-08

**Authors:** Linping Xu, Meimei Yan, Jianpeng Long, Mengmeng liu, Hui Yang, Wei Li

**Affiliations:** ^1^ Department of Research and Foreign Affairs, The Affiliated Cancer Hospital of Zhengzhou University and Henan Cancer Hospital, Zhengzhou, China; ^2^ Department of Hematology, The Affiliated Cancer Hospital of Zhengzhou University and Henan Cancer Hospital, Zhengzhou, China; ^3^ Academy of Medical Sciences of Zhengzhou University, Zhengzhou, China; ^4^ Department of Breast and Thyroid Surgery, Gansu Provincial Central Hospital, Lan Zhou, China; ^5^ Department of Gastroenterology, Zhengzhou University People’s Hospital and Henan Provincial People’s Hospital, Zhengzhou, China; ^6^ Department of Hematology, The First Affiliated Hospital of Zhengzhou University, Zhengzhou, China

**Keywords:** intrahepatic cholangiocarcinoma, immune cells, macrophages, SIRPα, MMP19

## Abstract

**Background:**

It has been shown that tumor-associated immune cells, particularly macrophages, play a fundamental role in the development and treatment response of intrahepatic cholangiocarcinoma (ICC). However, little is known about macrophages at the single cellular level of ICC patients.

**Methods:**

ScRNA-seq from Zhang et al. was used in the present study to identify the genes differentially expressed in ICCs. Furthermore, transcriptomic data from TCGA datasets, IHC and flowcytometry from our cohort were used to confirm the findings. Kaplan-Meier and TIDE scores were also used for prognostic analysis and ICB responses.

**Results:**

A significant number of macrophages were found in ICCs as compared to adjacent tissues. We then extracted, processed, and classified the macrophages from the ICCs and adjacent tissues into 12 clusters. Significantly, the macrophages from the ICC exhibited an immunosuppressed state in terms of both signature gene expression and functional enrichment. Furthermore, our results indicate that, of the 10 selective tumor-promoting genes of macrophages, only MMP19 and SIRPα can predict ICB responses in ICCs. Although a higher expression of MMP19 and SIRPα predict a poor prognosis for ICCs without immunotherapy after surgery, patients with high SIRPα expression were more sensitive to immunotherapy, whereas those with high MMP19 expression were not sensitive to immunotherapy. To define the mechanisms, we found that SIRPα^hi^ ICCs exhibited an increased enrichment KEGG pathway of leukocyte transendothelial migration and neutrophil extracellular trap formation. The increased immune cell infiltration will increase sensitivity to immunotherapy.

**Conclusion:**

Collectively, macrophages are critical to the immune status of ICCs, and MMP19 and SIRPα can predict prognosis and ICB responses for ICCs.

## Introduction

Intrahepatic cholangiocarcinoma (ICC) is an aggressive and invasive cancer that arises in the liver, which accounts for the second most common cause of liver cancer worldwide (1, 2). There have been an increasing number of ICC cases and deaths over the last decade, causing concern globally (3).The only potentially curative treatment for patients with resectable ICC is surgery (4, 5). Despite liver resections being undertaken as a curative procedure, the 5-year survival rate of patients with liver resections is disappointing, with a 20% to 35% survival rate (4).In addition, since the onset of ICC is so insidious, most patients by the time of diagnosis have already reached a stage of advanced or metastatic cancer, and only a small proportion of tumors are resectable (3, 6). Adjuvant therapies, such as systemic chemotherapy and radiotherapy, are still poorly defined and have been reported to be only modestly effective (7, 8). The clinical effectiveness of immunotherapy and targeted therapies for patients with ICC remains ineffective, despite significant progress in both fields (9). It is therefore important to understand the immune microenvironment of ICCs for effective diagnosis and treatment.

Tumor tissue consists of tumor cells and tumor stroma. The tumor stroma is composed of both immune and non-immune stromal cells, which together form a complex microenvironment that promotes tumor growth (10). Drug development targeting the tumor microenvironment (TME) is a promising approach to treating many forms of cancer, and it is especially relevant to ICCs (11).In 2020, Zhang et al. first described the cellular dissection of ICCs and showed that the cellular landscape of ICCs exhibits a high level of diversity of malignant, immune and stromal cells (12). In their study, Zhang et al (12). characterized distinct populations of immune and stromal cells, in particular tumor-infiltrating lymphocytes (TILs) and cancer-associated fibroblasts (CAFs) in the context of a wide inter- and intra-tumor heterogeneity that also involve malignant cholangiocytes, as has been anticipated by several studies conducted on ICCs (13). Miao Su et al. established nine-prognosis-related-genes (PRGs) by reanalyzing the ScRNA-seq (14). These genes included SLC16A3, BNIP3L, TPM2, CLEC11A, EREG, PMAIP1, CEBPB, A2M, and TUBA1B, which are correlated with ICC prognosis (14). Studies show that macrophages are primarily responsible for regulating immune functions, hematopoiesis, metabolism, tissue repair, and maturation of embryonic tissue (15–19). However, the prognostic genes expressed by macrophages in ICCs, still has to be further investigated.

In this study, single-cell sequencing data, transcriptome sequencing data from the TCGA database, and flow cytometric analysis and immunohistochemical validation of ICC from our own unit were used to investigate in depth the prognostic role of altered immune cell subpopulations, particularly genes heterogeneously expressed by macrophages. In ICCs, we observed an increase in macrophages that are crucial for ICC development. An analysis of differentially expressed genes revealed that the macrophages in ICCs predominantly exhibited an immunosuppressive phenotype, whereas macrophages in tissues adjacent to the cancer predominantly exhibited an immune activating phenotype. Furthermore, MMP19^hi^ ICCs predict poorer response to ICB treatment and shorter DFS and OS compared with MMP19^low^ ICCs. Although the patients with SIRPα^hi^ ICCs respond better to ICB treatment, those with SIRPα^hi^ ICCs without immunotherapy had a shorter DFS and OS compared with SIRPα^low^ ICCs, which indicates that patients with SIRPα^hi^ should receive ICB treatment. Transcriptome sequencing explains the underlying molecular mechanisms of these two different biomarkers in predicting differences in ICB treatment response. Our study highlights that macrophage plays an important role in the progression of ICCs, and targeting macrophage therapy may be a new and effective approach in immunotherapy.

## Materials and methods

### ScRNA-seq database

The scRNA-seq datasets of ICC cells and paracancer tissue cells were acquired from the Gene Expression Omnibus (GEO) database (GSE138709, GSE142784), and the 10X Genomic scRNA-seq data was acquired from five ICC samples and three adjacent tissues reported by Zhang et al. (12).The information regarding the cell preparation and single-cell transcriptome profiling is described in the original paper (12).

### Quality control and data processing

We combined single-cell data from ICC patients and healthy donors using the merge function found in version 3.2.2 of the Seurat R package, as previously described (20). Briefly, the cells that have unique feature counts of > 3,000, < 200 and ≥ 10% mitochondrial counts were filtered. The merged dataset was normalized using the Seurat “NormalizedData” function, with a global scaling normalization method “LogNormalize,” and this was multiplied by a scale factor (10,000 by default). It was then scaled by performing the Seurat “ScaleData” function, with regression of the variation of “nCount_RNA” and “percent.mt.” Performing the Seurat “JackStrawPlot” and “ElbowPlot” functions aided in the selection of suitable dimensionality. Dimension reduction analysis was performed with the Seurat “RunPCA” function, and non-linear dimensional reduction was performed with the Seurat “RunUMAP” function. The tSNE analysis was used for dimension reduction to determine the cell populations and for visualization of the gene expression (14). The FindClusters function was performed with a resolution of 0.5, and the RunTSNE function was used to generate clusters. The FindAllMarkers function (arguments: min.pct = 0.25, logfc.threshold = 0.25) was used to find markers by comparing each cluster to all others, and different genes between two identities were identified with the FindMarkers function. The feature plot and heatmap visualization of gene expression were generated using the Seurat functions FeaturePlot and DoHeatmap, respectively (14). Clusters consisting of macrophages were extracted and processed again as described above, and each macrophage type was further divided into subclusters. Marker genes of each macrophage type were identified by comparing ICC subclusters with adjacent subclusters, and adjustment of P-value (adjPval) < 0.05 was regarded as the cutoff criteria. The marker genes of each macrophage type were incorporated as differentially expressed genes (DEGs). Finally, macrophages from tumor tissue were extracted and processed again as described above. Differences in pathway activity were analyzed by a gene set variation analysis (GSVA) (12).

### Single cell suspension preparation and flow cytometry

The freshly resected surgical ICCs and adjacent tissue samples from our cohort were immediately washed with phosphate-buffered saline (PBS) and processed to generate single cell suspensions for the subsequent flowcytometry. Tissue digestion was performed in 15 ml tubes containing 10 ml pre-warmed RPMI 1640 (ThermoFisher Scientific) with trypsin, 1 mg/ml type IV collagenase (Sigma) and 10 U/μl DNase I (Roche) at 37°C for 30 minutes. The reaction was deactivated with 10% FBS. Cell suspensions were filtered using a 70 μM filter and then centrifuged at 500 rpm at 4°C for 6 min to pellet dead cells and red blood cells. The cells were washed twice and suspended in PBS with 0.5% bovine serum albumin (BSA, Sigma). Thereafter, the concentration of the single cell suspension was calculated, and 5x10^6^ cells were taken for subsequent flow cytometry assays, as previously described (18, 21, 22). The flowcytometry method was used to analyze the macrophage percentage by staining with anti-human APC-Cy7-CD45 (Biolegend, Cat#:304014, 0.1μl/10^6^ cells), anti-human Percp-CD14 (Biolegend, Cat#: 325632, 0.1μl/10^6^ cells) and anti-human PE-Cy7-CD16 (Biolegend, Cat#: 302015,0.1μl/10^6^ cells). After blocking, cells from ICC and adjacent tissues were stained with anti-human APC-Cy7-CD45, anti-human Percp-CD14 and anti-human PE-Cy7-CD16 on ice for 30 minutes in the dark. After staining, the cells were washed once with PBS plus 0.5% BSA and 2 mM EDTA. DAPI were used to gate out dead cells, and then the cells were resuspended in PBS plus 0.5% BSA and 2mM EDTA and run on a BD Air III (BD bioscience). Flow Jo software (BD) was used to analyze the data.

### ICC samples, follow-up and survival analysis

Twenty ICC and 20 adjacent tissue samples were obtained from patients who underwent curative resection of ICCs between October 1, 2016 and October 1, 2019 at the Affiliated Cancer Hospital of Zhengzhou University. This study was approved by the Ethics Committee at the Affiliated Cancer Hospital of Zhengzhou University. All methods and procedures associated with this study were conducted in accordance with the Good Clinical Practice guidelines and aligned with the ethical principles of the Declaration of Helsinki as well as local laws. All enrolled patients were pathologically diagnosed with ICCs and did not receive any anti-cancer treatments before surgery. Follow-up and survival analysis of ICC patients were consistent with methods used in previous studies (23, 24). After surgery, the patients were checked regularly. During the first two years, follow-up evaluations were measured every 3 months. During the 2 to 5 year period after surgery, the follow-up tests of ICC patients were examined every 6 months, and, beyond 5 years, they were measured every year. The follow-up tests included complete blood examinations, tumor biomarkers and chest and abdominal computed tomography scans. If follow-up evaluations revealed metastatic disease and/or local recurrences, other therapies were applied, including conventional therapies (surgery, chemotherapy, and radiotherapy) as well as targeted and immunotherapy. Disease-free survival (DFS) was calculated from the date of surgery to the time of recurrence or metastasis, and patients who were alive and in a stable state were censored at the time of last contact (25–27). Overall survival (OS) was calculated from the date of surgery to the time of death, and patients who were alive at the time of last contact were censored (25–27). DFS and OS were calculated using the Kaplan–Meier analysis. The final follow-up was performed on January 1, 2022.

### IHC staining and quantitative analysis

Formalin-fixed and paraffin-embedded sections of intrahepatic cholangiocarcinoma tissue (N=20) and paracancerous tissue (N=20, 5μm thick) from our cohort were dewaxed and rehydrated. Antigen retrieval was performed by heating the slides in 10 mM Tris buffer with 1 mM EDTA (pH 9) in a steamer for 20 min. Inhibition of endogenous peroxidase activity was achieved by immersion in 3% H_2_O_2_ for 5 minutes. After washing the tissue with Tris-buffered saline (TBS) containing Tween, endogenous biotin was inhibited through sequential incubation with 0.1% anti-biotin protein and 0.01% biotin (Dako, Glostrup, Denmark), respectively, at room temperature for 10 min. Other non-specific binding sites were blocked with 3% skimmed milk powder at room temperature for 30 minutes. The ICC and paracancerous tissue sections were incubated with the monoclonal mouse antibody anti-human CD68 (Abcam, Cat# ab213363), SIRPα (Abcam, Cat# ab260039), and MMP19 (Abcam, Cat# ab53146) at 4 °C for one night. Subsequently, the sections were serially rinsed and incubated with secondary antibodies. The immunohistochemical staining was evaluated independently by two experienced pathologists, who were blinded to the patients’ clinical characteristics and outcomes. H-score was used to quantify the expression of CD68, SIRPα, and MMP19 as previously described (28). The median was selected as the cutoff value for high or low CD68, SIRPα and MMP19 expression.

### TCGA datasets

The Limma package (version 3.40.2) of R software was used to study the differential expression of mRNAs (29).The adjusted *P*-value was analyzed to correct for false positive results in TCGA or GTEx. “Adjusted *P* < 0.05 and Log_2_ (Fold Change) > 1 or Log_2_(Fold Change) < −1” were defined as the thresholds for the screening of differential mRNA expression. Raw counts of RNA-sequencing data of 36 ICC patients from TCGA datasets were used to analyze the differentially expressed genes, based on the high and low expression of selective genes. To further confirm the underlying function of the potential targets, the data was analyzed *via* functional enrichment. The Kyoto Encyclopedia of Genes and Genomes (KEGG) Enrichment Analysis is a practical resource for the analytical study of gene functions and associated high-level genome functional information. To better understand the carcinogenesis of mRNA, the ClusterProfiler package (version: 3.18.0) of R was employed to analyze the enrichment of the KEGG pathway as our previous described (26).

### ICB responses analysis

Raw counts of RNA-sequencing data and corresponding clinical information from 36 ICC patients were obtained from TCGA dataset (https://portal.gdc.cancer.gov/), with methods of acquisition and application that comply with the guidelines and policies. Potential ICB response was predicted with a TIDE algorithm, as our and other authors previously described (26, 30). Using gene expression markers, TIDE provides insight into two different mechanisms of tumor immune escape, namely dysfunction of tumor-infiltrating cytotoxic T lymphocytes (CTL) and exclusion of CTL by immunosuppressive factors. It has been shown that high TIDE scores are associated with poor immunocheckpoint blockade therapy (ICB) efficacy and short survival following ICB treatment (26). Therefore, TIDE score is a novel method for predicting ICB responses.

### Statistical analysis

GraphPad Prism 9.0 software (GraphPad Software, Inc.) was used to perform the statistical analysis. Quantification of CD68, SIRPα, and MMP19 density and CD14 and CD16 percentage were analyzed *via* a *t*-test. The DFS and OS were calculated using the Kaplan-Meier estimator. *P* < 0.05 was used to indicate a statistically significant difference for all data.

## Results

### ScRNA-seq characterization of the immunomicroenvironment ofhuman ICCs

**Figure 1:**
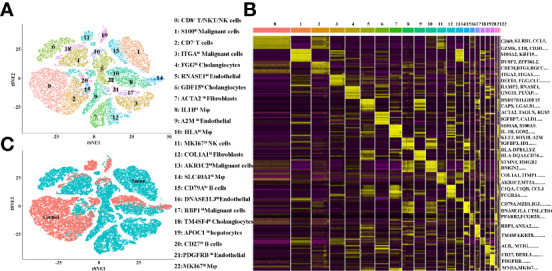
**(A)** tSNE plots for the cell type identification of high-quality single cells from ICC and adjacent tissues. **(B)** Heatmap showing the top DEGs in each cell type. **(C)** tSNE plots for cells from ICC (tumor) and adjacent (control) tissues.

The TME is formed by tumor cells, tumor-associated fibroblasts, and the surrounding tissues, immune cells, blood vessels and extracellular matrix, and other elements. In the present study, we investigated the TME of ICC patients by 10X Genomics sequencing from the GEO database (12). The quality control chart shows the detected gene number range, UMI count for each cell and the percentage of mitochondrial genes (**Supplementary Figures 1A–G**). In the original paper, the cells were classified into 10 distinct cell types, using known marker genes (12). To further cluster the cells precisely, the samples were successfully classified into 23 clusters with the t-distributed stochastic neighbor embedding (t-SNE) algorithm. Based on the expression of canonical and functional marker genes, these clusters included the following (**Figure 1A** and **Supplementary Table 1**):

- Cluster 0 (CD8^+^T/NKT/NK cells, marked with CD3E, CD8A, GZMA, GZMH, CD69, KLRB1, IL32 and CCL5, **Supplementary Figure 2**),
- Cluster 1 (S100^hi^ malignant cells, marked with S100A2, AKR1C2, S100A6, S100A10, S100A16, S100A14 and KRT7, **Supplementary Figure 8**),
- Cluster 2 (CD7^+^ T cells, malignant cells, marked with DUSP2, CREM, CD7 and CXCR4, **Supplementary Figure 2**),
- Cluster 3 (ITGA^hi^ malignant cells, marked with PMEPA1, ITGA2, ITGA3 and KRT19 **Supplementary Figure 8**),
- Cluster 4 (FGG^hi^ cholangiocytes, marked with FGG, DEFB1, CLU, TM4SF4 and AKR1C3, **Supplementary Figure 7**),
- Cluster 5 (RNASE1^hi^ endothelial cells, marked with RAMP2, RNASE1, CCL2 and CLEC14A **Supplementary Figure 6**),
- Cluster 6 (GDF15^hi^ cholangiocytes, marked with GDF15, CAPS and ANXA4, **Supplementary Figure 7**),
- Cluster 7 (ACTA2^hi^ fibroblasts, marked with ACTA2, TAGLN, MYL9 and NDUFA4L2, **Supplementary Figure 5**),
- Cluster 8 (IL1B^hi^ macrophages, marked with S100A8, S100A9, IL1B, AIF1, CD68 and LYZ, **Supplementary Figure 4**),
- Cluster 9 (A2M^hi^ endothelial cells, marked with CLDN5, A2M, VWF, CD34 and RAMP2, **Supplementary Figure 6**),
- Cluster 10 (HLA^hi^ macrophages, marked with HLA-DPB1, HLA-DQA1, HLA-DQA1, HLA-DRB1 and CD74, **Supplementary Figure 4**),
- Cluster 11 (MKI67^hi^ NK cells, marked with MKI67, HMGB2, GZMA, NKG7 and GZMB, **Supplementary Figure 3**),
- Cluster 12 (COL1A1^hi^ fibroblasts, marked with COL1A1, COL3A1, DCN, IGFBP7 and CCL2, **Supplementary Figure 5**),
- Cluster 13 (AKR1C2^hi^ malignant cells, marked with AKR1C2, KRT7 and HMGA1, **Supplementary Figure 8**),
- Cluster 14 (SLC40A1^hi^ macrophages, marked with C1QA, C1QB, C1QC, APOE, FCGR3A and SLC40A1, **Supplementary Figure 4**),
- Cluster 15 (CD79A^hi^ B cells, marked with CD79A, MZB1, IGJ, DERL3 and CD79B, **Supplementary Figure 3**),
- Cluster 16 (DNASE1L3^hi^ endothelial cells, marked with DNASE1L3, CD36, PPAP2B, AKAP12 and RAMP3, **Supplementary Figure 6**),
- Cluster 17 (RBP1^hi^ malignant cells, marked with RBP1, FN1, ANXA2 and KRT7, **Supplementary Figure 8**),
- Cluster 18 (TM4SF4^hi^ cholangiocytes, marked with TM4SF4, ANXA4 and DEFB1, **Supplementary Figure 7**),
- Cluster 19 (APOC1^hi^ hepatocytes, marked with ALB, MT1G and APOC1, **Supplementary Figure 7**),
- Cluster 20 (CD27^hi^ B cells, marked with SLAMF7, CD27, CD79A and MZB1, **Supplementary Figure 3**),
- Cluster 21 (PDGFRB^hi^ endothelial cells, marked with NDUFA4L2, PDGFRB, RAMP2 and TAGLN, **Supplementary Figure 6**),
- Cluster 22 (MKI67^hi^ macrophages, marked with MNDA, MKI67 and SPI1, **Supplementary Figure 3**).

More marker genes of each cluster are demonstrated in **Figure 1B** and **Supplementary Table 1**, which confirmed the accuracy of cell identity. In addition, the cells derived from the ICC or control tissues are shown in **Figure 1C**. This data demonstrated that Clusters 0, 2, 10, 11, 16, 18 and 19 were from the control tissues, and, in contrast, Clusters 1, 3, 4, 5, 6, 7, 8, 9, 12, 13, 14, 15, 17, 20, 21 and 22 were from the cancer tissues.

### Macrophages increased in the microenvironment of human ICCs

**Figure 2:**
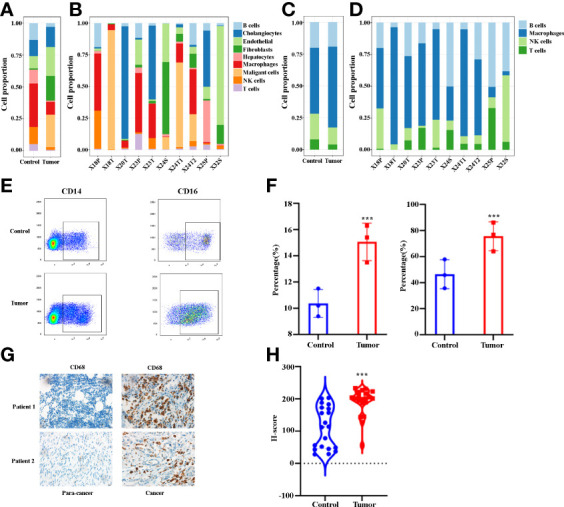
**(A)** Bar plots showing the proportion of cell types in tumor and control samples. **(B)** Bar plots showing the proportion of cell types in each sample. **(C)** Bar plots showing the proportion of immune cell types in tumor and control samples. **(D)** Bar plots showing the proportion of immune cell types in each sample. **(E)** The representative flowcytometry images of CD14 and CD16 expression in CD45^+^ cells in control and tumor samples. **(F)** Quantitative analysis revealed CD14 and CD16 expression in tumor and control tissues (n = 3). **(G)** The representative IHC images of CD68 expression in control and tumor samples. **(H)** Quantitative analysis show CD68 expression in tumor and control tissues (n = 20). ***P < 0.001.

In order to better understand immune cell infiltration in human ICC, we first analyzed the cell proportion of all cell types in the control and the tumor tissue. **Figure 2A** demonstrates that B cells, hepatocytes, macrophages, NK cells and T cells in tumor samples decreased compared with the control samples. The proportion of each cell type varied greatly by sample (**Figure 2B**), which suggests the heterogeneity of the ICC samples. We then analyzed the immune cell proportion in the control and tumor tissue. As shown in **Figure 2C**, macrophages are the only immune cells to increase. Remarkably, the macrophage subclusters were highly patient-specific (**Figure 2D**), suggesting the heterogeneity of macrophages in ICC patients. Thereafter, we investigated the changes in macrophages in ICC by staining CD14 and CD16. **Figure 2E** shows representative flowcytometry images of CD14 and CD16 of paracancer and cancer tissues. A quantitative analysis revealed that the CD14^+^ and CD14^+^CD16^+^ increase significantly (**Figure 2F**). We next examined the total macrophage percentage in the paracancer and cancer tissues by staining total macrophage marker CD68 by IHC. **Figures 2G, H** demonstrate that CD68 increases significantly in ICC patients. Collectively, our findings demonstrate that macrophages, especially CD14^+^CD16^+^ subclusters, are increased in the microenvironment of ICCs.

### Immunosuppressive tumor-associated macrophages enriched in human ICC tumors

**Figure 3:**
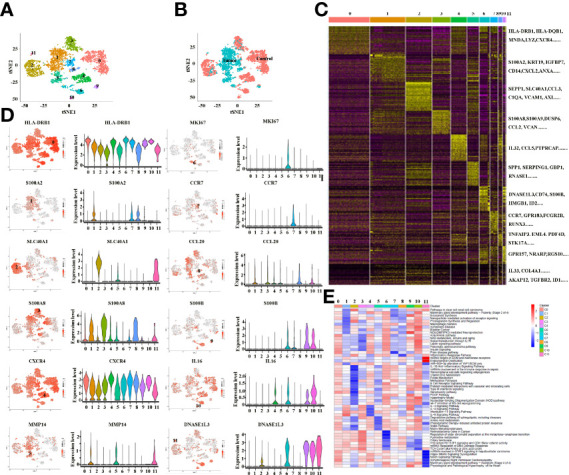
**(A)** tSNE plots for macrophage subpopulations from ICC and adjacent tissues. **(B)** tSNE plots for macrophages from ICC (tumor) and adjacent (control) tissues. **(C)** Heatmap showing the top DEGs in each macrophage subpopulation. **(D)** tSNE plots, color-coded (gray to red) for the expression of marker genes for each macrophage subpopulation, as indicated (left); Violin plots of marker genes for each macrophage subpopulation, as indicated (right); **(E)** Differences in pathway activity (scored per cell by GSVA) in 12 macrophage cell subclusters in tumor and control tissues.

Tumor-infiltrating immune cells are highly heterogeneous and have been shown to play important roles in immune evasion and the response to immunotherapy treatment (31). In the present study, the total macrophages from cancer and paracancer tissue were extracted, and they exhibited 12 distinct subclusters (**Figure 3A**), The tSNE plot shows the subcluster distribution among the sample pathology (**Figure 3B**). As illustrated in **Figures 3A, B**, Clusters 1, 5 and 9 were defined from cancer tissues, while the other clusters were from paracancer tissue. A heatmap of the top 30 marker genes for each cluster is shown in **Figure 3C**. All marker genes of these 12 clusters are demonstrated in **Supplementary Table 2**. According to the differentially expressed genes, the macrophages were designed as Cluster 0: HLA^+^, Cluster 1: S100A2^+^, Cluster 2: SLC40A1^+^, Cluster 3: S100A8^+^, Cluster 4: CXCR4^+^, Cluster 5: MMP14^+^, Cluster 6: MKI67^+^, Cluster 7: CCR7^+^, Cluster 8: CCL20^+^, Cluster 9: S100B^+^, Cluster 10: IL16^+^ and Cluster 11: DNASE1L3^+^ (**Figure 3D** and **Supplementary Table 2**). In addition, we found that macrophage subpopulations (clusters 1, 5, 7, 8, and 9) from cancer tissues expressed a certain number of immunosuppressive genes, such as VEGFA, MMP14, MMP19, S100A2, APOE, HMOX1, SLAMF7, SIRPα, LAG3 and IL18. In contrast, macrophage subpopulations (clusters 0, 2, 3, 4, 6, 10 and 11) from paracancer tissues are characterized by the prominent expression levels of activated markers such as HLA-DRB1, HLA-DQB1, HLA-DQA1, HLA-DPB1, HLA-DPA1, CD74, MNDA, CXCR4, CCR7, GPR183, IL32, CCL5 and IL16. Based on the marker genes, the GO terms enriched in Clusters 1, 5 and 9 were related to IL-3, 4, 9 and 11 signaling pathways, including the inflammatory response pathway, macrophage markers, NO/cGMP/PKG mediated neuroprotection, signal transduction through IL1R, insulin signaling, the IL10 anti-inflammatory signaling pathway, the PDGF pathway and matrix metalloproteinases (**Figure 3E**). In combination, these results reveal that the macrophages in cancer tissues exhibit highly immunosuppressive characteristics and suggest that manipulation of macrophages is potentially a novel therapeutic strategy for ICCs.

### Heterogeneity of macrophages in cancerous ICC tissues

**Figure 4:**
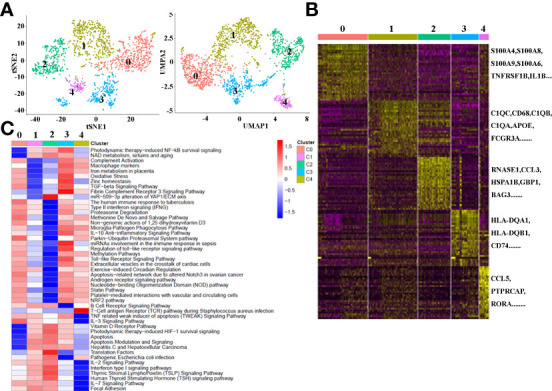
**(A)** tSNE plots for macrophage subpopulations from ICC tissue. **(B)** Heatmap showing the top DEGs in each macrophage subpopulation. **(C)** Differences in pathway activity (scored per cell by GSVA) in five macrophage cell subclusters in tumor tissue.

**Figure 5:**
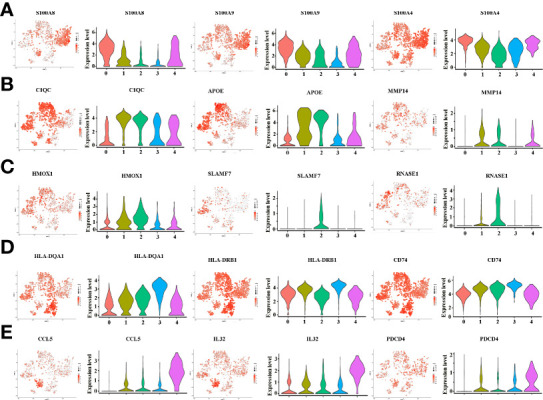
**(A)** tSNE plots, color-coded (gray to red) for the expression of marker genes for macrophage subpopulation 0, as indicated (left); Violin plots of marker genes for macrophage subpopulation 0, as indicated (right). **(B)** tSNE plots, color-coded (gray to red) for the expression of marker genes for macrophage subpopulation 1, as indicated (left); Violin plots of marker genes for macrophage subpopulation 1, as indicated (right). **(C)** tSNE plots, color-coded (gray to red) for the expression of marker genes for macrophage subpopulation 2, as indicated (left); Violin plots of marker genes for macrophage subpopulation 2, as indicated (right). **(D)** tSNE plots, color-coded (gray to red) for the expression of marker genes for macrophage subpopulation 3, as indicated (left); Violin plots of marker genes for macrophage subpopulation 3, as indicated (right). **(E)** tSNE plots, color-coded (gray to red) for the expression of marker genes for macrophage subpopulation 4, as indicated (left); Violin plots of marker genes for macrophage subpopulation 4, as indicated (right).

There is growing recognition that macrophage reprogramming, rather than blanket depletion, could be a superior option to utilize macrophages in cancer therapy. Therefore, it is necessary to identify the heterogeneity of macrophages in cancerous tissues of ICC patients. Macrophages were extracted from tumor tissue and reanalyzed. **Figure 4A** demonstrates five subpopulations of macrophages in tumors from the tSNE and UMAP analyses. A heatmap of the top 30 marker genes for each cluster is shown in **Figure 4B**. According to the differentially expressed marker genes, the macrophages in ICC tissues were designed as Cluster 0: S100A^+^ macrophages (**Figure 5A**), Cluster 1: C1QC^+^ macrophages (**Figure 5B**), Cluster 2: FCGR3A^+^ macrophages (**Figure 5C**), Cluster 3: HLA^+^ macrophages (**Figure 5D**) and Cluster 4: CCL5^+^ macrophages (**Figure 5E**). All the differentially expressed marker genes in each cluster are shown in **Supplementary Table 3**. Based on the marker genes, we analyzed the GO terms to compare the significant differences in the pathways in each cluster. The GO terms enriched in Cluster 0 included the human immune response to tuberculosis, the parkin-ubiquitin proteasomal system pathway, the methionine *de novo* and salvage pathway and the IL-3 signaling pathway. The GO terms enriched in Cluster 1 included focal adhesion, the IL-7 signaling pathway and the B cell receptor signaling pathway. The GO terms enriched in Cluster 2 included NAD metabolism, sirtuins, aging and miR-509-3p alteration of YAP1/ECM axis. Vitamin D receptor pathway, photodynamic therapy-induced HIF-1 survival signaling, translation factors, and interferon type I signaling pathways. The GO terms enriched in Cluster 3 included photodynamic therapy-induced NF-kB survival signaling, oxidative stress, zinc homeostasis, the TGF-beta signaling pathway, the fibrin complement receptor 3 signaling pathway, the microglia pathogen phagocytosis pathway, the IL-10 anti-inflammatory signaling pathway, miRNAs involvement in the immune response in sepsis and the Toll-like receptor signaling pathway. The GO terms enriched in Cluster 4 included the T cell receptor pathway during staphylococcus aureus infection, methylation pathways and extracellular vesicles in the crosstalk of cardiac cells (**Figure 4C**). In conclusion, our findings revealed different macrophage subpopulations and a highly heterogeneous immune microenvironment that improves our understanding of ICC pathogenesis.

### Immune checkpoint blockade responses analysis based on selective tumor-promoting genes of TAMs in ICC

**Figure 6:**
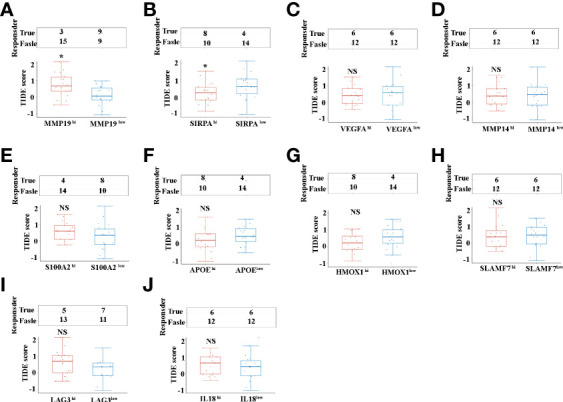
**(A)** The ICB response score (TIDE score) between MMP19^hi^ and MMP19^low^ ICC from 36 TCGA samples. **(B)** The ICB response score (TIDE score) between SIRPA^hi^ and SIRPA^low^ ICC from 36 TCGA samples. **(C)** The ICB response score (TIDE score) between VEGFA^hi^ and VEGFA^low^ ICC from 36 TCGA samples. **(D)** The ICB response score (TIDE score) between MMP14^hi^ and MMP14^low^ ICC from 36 TCGA samples. **(E)** The ICB response score (TIDE score) between S100A2^hi^ and S100A2^low^ ICC from 36 TCGA samples. **(F)** The ICB response score (TIDE score) between APOE^hi^ and APOE^low^ ICCs from 36 TCGA samples. **(G)** The ICB response score (TIDE score) between HMOX1^hi^ and HMOX1^low^ ICC from 36 TCGA samples. **(H)** The ICB response score (TIDE score) between SLAMF7^hi^ and SLAMF7^low^ ICC from 36 TCGA samples. **(I)** The ICB response score (TIDE score) between LAG3^hi^ and LAG3^low^ ICC from 36 TCGA samples. **(J)** The ICB response score (TIDE score) between IL18^hi^ and IL18^low^ ICC from 36 TCGA samples. *P <0.05, NS, no significant difference.

Programmed death 1 (PD-1), programmed death-ligand 1 (PD-L1) and cytotoxic T-lymphocyte-associated protein 4 (CTLA4) monoclonal antibodies are currently the most prominent targets for immunotherapy in ICCs, yet approximate one-third of patients have been found to respond to immunotherapy in clinical use (9). Tumor immune dysfunction and exclusion (TIDE) is a sufficient approach to predict the ICB response (30).We investigated the ICC patients’ response to the ICB, based on selective immunosuppressive genes of TAMs from TCGA dataset. Our results indicated that, of the selective 10 tumor-promoting genes of TAMs, only MMP19 (**Figure 6A**), and SIRPα (**Figure 6B**) can predict ICB response in ICC patients. Interestingly, lower ICB responses are predicted for MMP19^hi^ ICC patients, compared with MMP19^low^ patients (**Figure 6A**). In contrast, higher ICB responses are predicted for SIRPα^hi^ ICC patients, compared with SIRPα^low^ patients (**Figure 6B**). Furthermore, there are no significant ICB response predictions with high and low TIDE score groups of VEGFA (**Figure 6C**), MMP14 (**Figure 6D**), S100A2 (**Figure 6E**), APOE (**Figure 6F**), HMOX1 (**Figure 6G**), SLAMF7 (**Figure 6H**), LAG3 (**Figure 6I**) and IL18 (**Figure 6J**).

### The expression of MMP19 and SIRPα significantly increased in ICC patients and predict poor overall survival time with high expression

**Figure 7:**
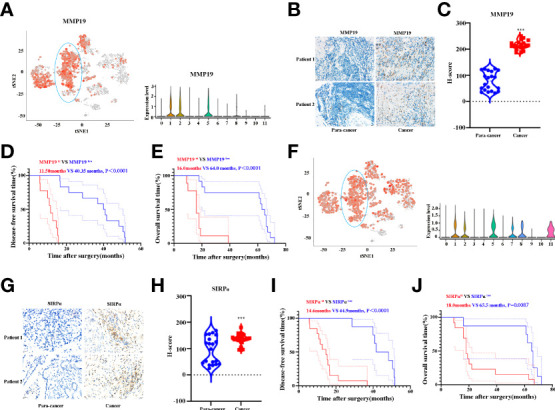
**(A)** tSNE plots, and Violin plots for the expression of MMP19 for macrophage subpopulations. **(B)** The representative IHC images of MMP19 expression in control and tumor samples. **(C)** Quantitative analysis revealed MMP19 expression in tumor and control tissues (n = 20). **(D)** The DFS curves of ICC patients based on the high and low expression of MMP19. **(E)** The OS curves of ICC patients based on the high and low expression of MMP19. **(F)** tSNE plots, and Violin plots for the expression of SIRPα for macrophage subpopulations. **(G)** The representative IHC images of SIRPα expression in control and tumor samples. **(H)** Quantitative analysis revealed SIRPα expression in tumor and control tissues (N = 20). **(I)** The DFS curves of ICC patients based on the high and low expression of SIRPα. **(J)** The OS curves of ICC patients based on the high and low expression of SIRPα. ***P < 0.001.

Having shown that MMP19 and SIRPα can predict the ICB responses, we proceeded to confirm the expression of MMP19 and SIRPα by IHC between ICC and paracancer tissues. Twenty ICC and 20 paracancer tissue samples collected between July 1, 2016 and July 1, 2019 from The Affiliated Cancer Hospital of Zhengzhou University were tested for the expression of MMP19 and SIRPα. All the patients had undergone surgery and were not treated with immunotherapies during the treatment process until death. At the last contact (December 31, 2021), all the patients were deceased. **Figure 7A** indicated the expression of MMP19 on macrophage subclusters from ICC and paracancer tissue by Sc-RNA-seq. **Figures 7B, C** demonstrate that the expression of MMP19 increased significantly in ICC compared to the paracancer tissue. A Kaplan–Meier analysis indicated that MMP19^hi^ patients had a shorter DFS (**Figure 7D**) and OS (**Figure 7E**) compared with MMP19^low^ patients. Using the same method, we showed the expression of SIRPα on macrophage subclusters from ICC and paracancer tissue by Sc-RNA-seq (**Figure 7F**). And we confirmed that SIRPα increased significantly in ICC tissue (**Figures 7G, H**). The Kaplan–Meier analysis revealed that SIRPα^hi^ patients had a shorter DFS (**Figure 7I**) and OS (**Figure 7J**) than SIRPα^low^ patients. Collectively, higher expression of MMP19 and SIRPα predict a poor prognosis for ICC patients after surgery.

### Transcriptome analysis of ICC tissue based on the high and low expression of MMP19 or SIRPα

**Figure 8:**
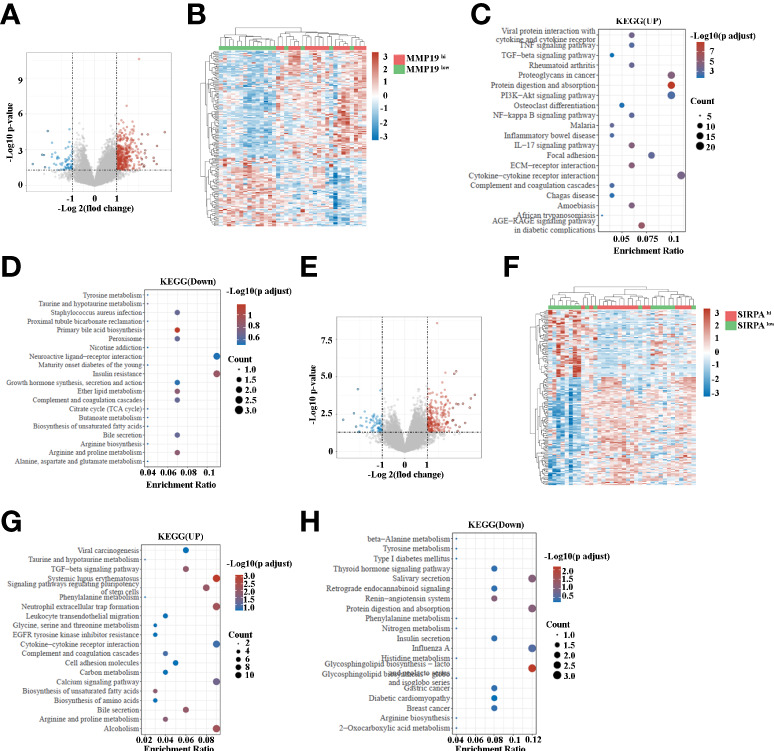
**(A)** Volcano map indicating the differentially expressed genes in MMP19^hi^ and MMP19^low^ groups of ICC patients. **(B)** Heatmap of differentially expressed genes in MMP19^hi^ and MMP19^low^ groups of ICC patients. **(C)** The upregulated KEGG pathways in the MMP19^hi^ ICC patients. **(D)** The downregulated KEGG pathways in the MMP19^hi^ ICC patients. **(E)** Volcano map indicating the differentially expressed genes in SIRPα^hi^ and SIRPα^low^ groups of ICC patients. **(F)** Heatmap of differentially expressed genes in SIRPα^hi^ and SIRPα^low^ groups of ICC patients. **(G)** The upregulated KEGG pathways in the SIRPα^hi^ ICC patients. **(H)** The downregulated KEGG pathways in the SIRPα^hi^ ICC patients.

Having shown that MMP19^hi^ expression predicts a poor response to ICB treatment, while SIRPα^hi^ expression predict a better response to ICB treatment in ICC patients, we analyzed the transcriptome data of MMP19^hi^ and MMP19^low^ ICC tissues. A total of 495 genes were distinguished as differentially expressed mRNAs, with 431 genes upregulated and 64 genes downregulated (**Figures 8A, B** and **Supplementary Table 4**). The selective upregulated genes in MMP19^hi^ patients included MMP12, MMP7, VCAM1, IL6, GAS7, CXCL5, CXCL13, CCL21, CCL18 and S100A1. **Figure 8C** shows the enrichment of KEGG pathways in the MMP19^hi^ group, including focal adhesion, ECM-receptor interaction, the IL17 signaling pathway, the TNF signaling pathway, the TGF-beta signaling pathway and cytokine-cytokine receptor interaction. The downregulated KEGG pathways in the MMP19^hi^ group included the primary bile acid biosynthesis, insulin resistance, bile secretion and biosynthesis of unsaturated fatty acids (**Figure 8D**). We next analyzed the transcriptome differences between SIRPα^hi^ and SIRPα^low^ ICCs with the same method. A total of 296 genes were distinguished as differentially expressed mRNAs, with 234 genes upregulated and 62 genes downregulated (**Figures 8E, F** and **Supplementary Table 5**). The selective upregulated genes in SIRPα^hi^ patients included IGFBP1, APOA2, ALB, TFR2, SLC23A1, CDH6, TNFSF15, APOB, GDF15, SLC17A1, SLC17A3, SLC28A1, SLC39A5, SLC27A2, CXCL8, SLC40A1, CXCL12, TGF-B2, CCL2 and CXCL6 (**Supplementary Table 5**). **Figure 8G** shows the enrichment of KEGG pathways in the SIRPα^hi^ group, including cell adhesion molecules, the calcium signaling pathway, biosynthesis of unsaturated fatty acids, bile secretion, biosynthesis of amino acids, leukocyte transendothelial migration and neutrophil extracellular trap formation. The downregulated KEGG pathways in the SIRPα^hi^ group included nitrogen metabolism, insulin secretion and histidine metabolism (**Figure 8H**).

## Discussion

A growing body of research has revealed that a deeper understanding of the TME, in particular the characterization of tumor-infiltrating immune cells, is essential for exploring key regulatory molecules in tumor development and immunotherapy (11, 32, 33). Using 10X genomic single-cell sequencing, Zhang et al. report the first complete subpopulation of cholangiocarcinoma immune microenvironment cells (12). The study identified six distinct subpopulations of fibroblasts, most notably vascular cancer-associated fibroblasts (vCAFs) that express microvascular signature genes. Therefore, the study focused on a novel cellular interaction between ICCs and vCAFs and identified potential targets for the treatment of ICC (12). Chen et al. (34) reanalyzed the Sc-RNAseq data and found that PNOC was mainly expressed by B cells in TME and could be an independent indicator for a better prognosis. LAIR2 was mainly expressed by Treg and partial CD8^+^/GZMB T cells, which could be an indicator of exhaustive T cell populations in TME. Both PNOC and LAIR2 were correlated with high immune infiltration levels in ICC patients (34). Miao Su et al. defined nine prognostic genes, including SLC16A3, BNIP3L, TPM2, CLEC11A, EREG, PMAIP1, CEBPB, A2M, and TUBA1B using the same Sc-RNAseq data (14). However, the roles of macrophages on the single-cell molecular profile in the prognosis of patients with cholangiocarcinoma are still unclear. In the present study, we used Sc-RNAseq data, bulk sequencing data of cholangiocarcinoma patients in TCGA database, flowcytometry analysis and IHC analysis for an in-depth investigation of the immune phenotypes and macrophage subpopulations and their heterogeneity and roles in the prognosis of ICCs.

Significantly, in the original study of Zhang et al. (12), they did not fully investigate the function of macrophages. In the present study, we first examined immune infiltration in ICCs. The percentage of macrophages in the total number of immune cells increased significantly. The increased macrophage infiltration in ICCs was also confirmed by IHC, which is consistent with our previous study (25). Molecularly, macrophages can be divided into two types, namely M1- and M2-like macrophages and these cells are involved in anti-tumor activity and immunosuppressive tumor promotion (35, 36). Using fresh ICC and paracancer tissue, we found that the percentage of CD14^+^CD16^+^ macrophages (M2-like) had significantly increased in ICCs. Previous studies indicated that CD14^+^CD16^+^ macrophages can suppress T-cell activation in MM patients (37). While, the “M1-M2” macrophage dichotomy is too simple to describe their complicated roles in the TME (38). Significantly, not all tumor-promoting TAMs have M2-like phenotypes, thus highlighting the importance of defining TAM states beyond the M1/M2 dichotomy (39). Single-cell sequencing technology is an important method to study TAM heterogeneity (39). In the present study, we classified macrophages originating from tumor and paracancerous tissue into 12 subpopulations using ScRNA-seq. Clusters 1, 5 and 9 are mainly from ICC tissue, which expressed a number of tumor-promoting genes, such as VEGFA (40), MMP14 (41, 42), MMP19 (43), S100A2 (44), APOE (45), HMOX1 (46), SLAMF7 (47), SIRPα (25, 48), LAG-3 (49), and IL-18 (50). These biomarkers are correlated with poor prognosis of many types of cancers. In contrast, macrophage subpopulations (Clusters 0, 2, 3, 4, 6, 7, 8, 10 and 11) from paracancer tissues were characterized by prominent expression levels of immune activated markers, such as HLA-related molecules (HLA-DRB1, HLA-DQB1, HLA-DQA1, HLA-DPB1, HLA-DPA1 and CD74) (51), MNDA (52), CXCR4 (53), CCR7 (54), GPR183 (55), IL32 (56), CCL5 (57), and IL16 (58). Despite these findings, macrophages are extremely heterogeneous in ICCs. There is still a subpopulation of macrophages that overexpress antigen-presentation-related molecules HLA-DQA1, HLA-DRB1 and CD74 in patients with ICC. These results suggest that a “one-size-fits-all” approach to treating ICC by deleting macrophages is not a good treatment option. Clinical studies have also confirmed that deleting macrophages through CSF1R antibodies has poor clinical responses (59). Furthermore, Gloria H.Y. Lin et al. reported that disrupting CD47-SIRPα pathway triggers phagocytosis of cancer cells by all macrophage subpopulations, especially M1 and M2c macrophages (60). O’Connell P et al. found that elevated SLAMF7 expression was associated with T-cell depletion (61). And Chen J et al. found that patients with high SLAMF7 expression responded better to CD47-SIRPα blockade treatment compared with low SLAMF7 expression (47). Our present study demonstrated that SIRPα and SLAMF7 highly expressed in ICCs compared with paracancer tissues. Collectively, these results suggest that ICC patients are sensitive to blocking CD47-SIRPα axis and that future clinical trials should be conducted to test this hypothesis.

ICB immunotherapy significantly improves the prognosis of patients with a wide range of tumors; however, only a minority of patients can benefit from ICB treatment (30). Several biomarkers or methods, such as PD-L1 (62), TMB (63), MSI (64) and MMR (65), have been reported to predict responses to ICB in multiple types of tumors. TIDE is a sufficient approach to predict the response to ICB (66). In the present study, we tested the 12 immunosuppressive genes in ICCs, and our results indicate that only MMP19 and SIRPα can predict the response to ICB. Interestingly, MMP19^hi^ ICCs showed poor responses to ICB treatment. In contrast, SIRPα^hi^ ICCs demonstrated improved responses to ICB treatment. In addition, survival analysis revealed that MMP19^hi^ or SIRPα^hi^ ICCs have a shorter OS. Transcriptome sequencing results showed that there are 431 upregulated genes and 64 downregulated genes in MMP19^hi^ ICCs. The upregulated enrichment of KEGG pathway in MMP19^hi^ ICCs included focal adhesion, ECM-receptor interaction, the IL17 signaling pathway, the TNF signaling pathway, the TGF-beta signaling pathway, and cytokine-cytokine receptor interaction, which can explain the poor response to ICB treatment, at least in part.

There is an interesting finding in ICCs with SIRPα^hi^ that are not treated with immunotherapy resulting in a shorter OS, indicating that SIRPα is a negative prognostic marker for ICCs. However, increased expression of SIRPα is associated with stronger responses to ICB therapy, indicating that SIRPα^hi^ ICCs require ICB therapy to extend OS. To explain the potential mechanisms, we performed a comparative analysis of transcriptomic data from ICC patients with high and low expression of SIRPα. The upregulated enrichment of KEGG pathways in the SIRPα^hi^ group included leukocyte transendothelial migration and neutrophil extracellular trap formation. In general, increased tumor-infiltrating leucocytes can lead to a poorer prognosis when the patient is not receiving immunotherapy (66), because most immune cells in SIRPα^hi^ ICCs have immunosuppressive phenotype. It is important to note, however, that the effectiveness of ICB treatment depends on the presence of tumor-infiltrating immune cells (67). CD47 acts as a “don’t eat me” signal that protects cells from phagocytosis by binding and activating its SIRPα receptor on macrophages (68). CD47 blockades or SIRPα fusion proteins direct targeting macrophages and significantly increase both innate and adaptive immunity against many types of cancer (68). Significantly, the effects of CD47 blockades or SIRPα fusion proteins depend on the presence of total macrophages (69). Both M1 and M2 macrophages are receptive to the action of CD47 monoclonal antibodies or SIRPα fusion proteins to exert their anti-tumor effects (60). Previous studies have indicated that both mouse and human TAMs express PD-1, and the expression levels increase during the growth of cancer in mouse models and high TNM staging in primary human cancers (70). PD-1^+^ TAMs exhibit an M2-like macrophage phenotype and show a significantly reduced phagocytic potency against cancer cells (70). However, inhibition of the interaction between PD-1 and PD-L1 *via* either a PD-1 or a PD-L1 blockade leads to an anti-tumor immune response in mice that lacks T cells, B cells and NK cells but has functional macrophages (70). In conclusion, increased macrophage infiltration indicates a poor prognosis in the absence of immunotherapy, and these patients may be more sensitive to ICB therapy.

However, our study has limitations. We have only validated the expression of some biomarkers and their relationships with the prognosis of small samples of ICCs and immunotherapy. Nevertheless, macrophages continue to play an important role in the progression of ICCs, and targeting macrophage therapy may be a new and effective means of immunotherapy. Last, independent studies with larger sample size are required to confirm the findings of this study.

## Data availability statement

The original contributions presented in the study are included in the article/**Supplementary Material**. Further inquiries can be directed to the corresponding author.

## Ethics statement

This study was reviewed and approved by The Affiliated Cancer Hospital of Zhengzhou University. The patients/participants provided their written informed consent to participate in this study.

## Author contributions

WL and LX designed, wrote, and edited the manuscript; analyzed the data; and finished the figures. HY, JL revised the manuscript. MY and ML analyzed the data. All authors contributed to the article and approved the submitted version.

## Funding

This study was supported by Key Research Projects of Henan Higher Education Institutions (21A320049) and Henan Province Medical Science and Technology Research Project (SBGJ202102063).

## Acknowledgments

We thank all of the authors listed in this manuscript.

## Conflict of interest

The authors declare that the research was conducted in the absence of any commercial or financial relationships that could be construed as a potential conflict of interest.

## Publisher’s note

All claims expressed in this article are solely those of the authors and do not necessarily represent those of their affiliated organizations, or those of the publisher, the editors and the reviewers. Any product that may be evaluated in this article, or claim that may be made by its manufacturer, is not guaranteed or endorsed by the publisher.

## Abbreviations

CAFs, Cancer-associated fibroblasts; CTLA4, cytotoxic T-lymphocyte-associated protein 4; GDF15, Growth differentiation factor 15; GEO, Gene Expression Omnibus; ICC, Intrahepatic cholangiocarcinoma; PD-1, Programmed death 1; PD-L1, programmed death-ligand 1; QC, Quality control; TILs, Tumor-infiltrating lymphocytes; TME, Tumor microenvironment.
