# Supplementary figures and images for: Identification of macrophage correlated biomarkers to predict the prognosis in patients with intrahepatic cholangiocarcinoma
Source: Front Oncol. 2022 Sep 8;12:967982. doi: 10.3389/fonc.2022.967982 (PMC9497456; doi:10.3389/fonc.2022.967982)

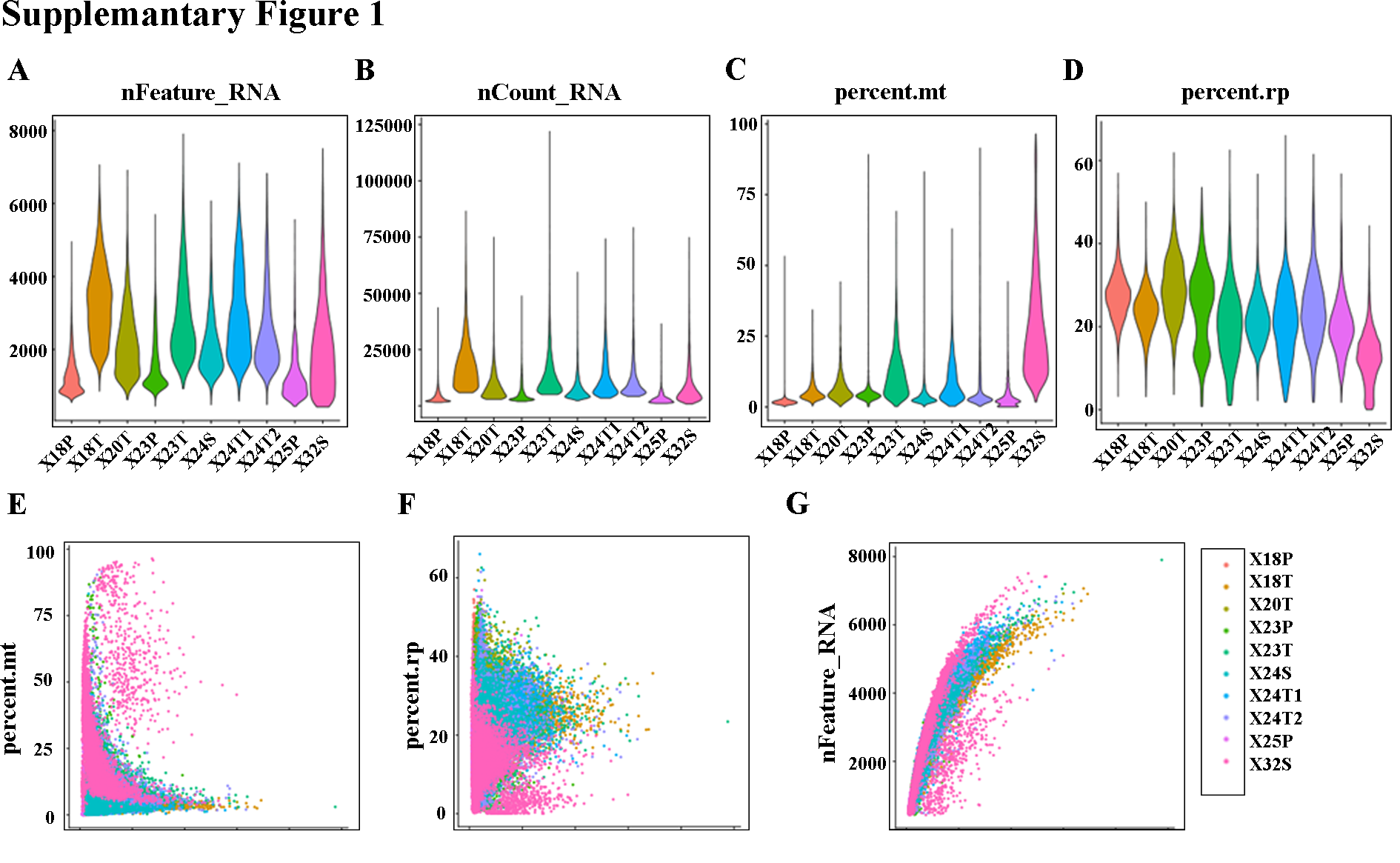

Supplement: Supplementary file 1 [file Image_1.tif]

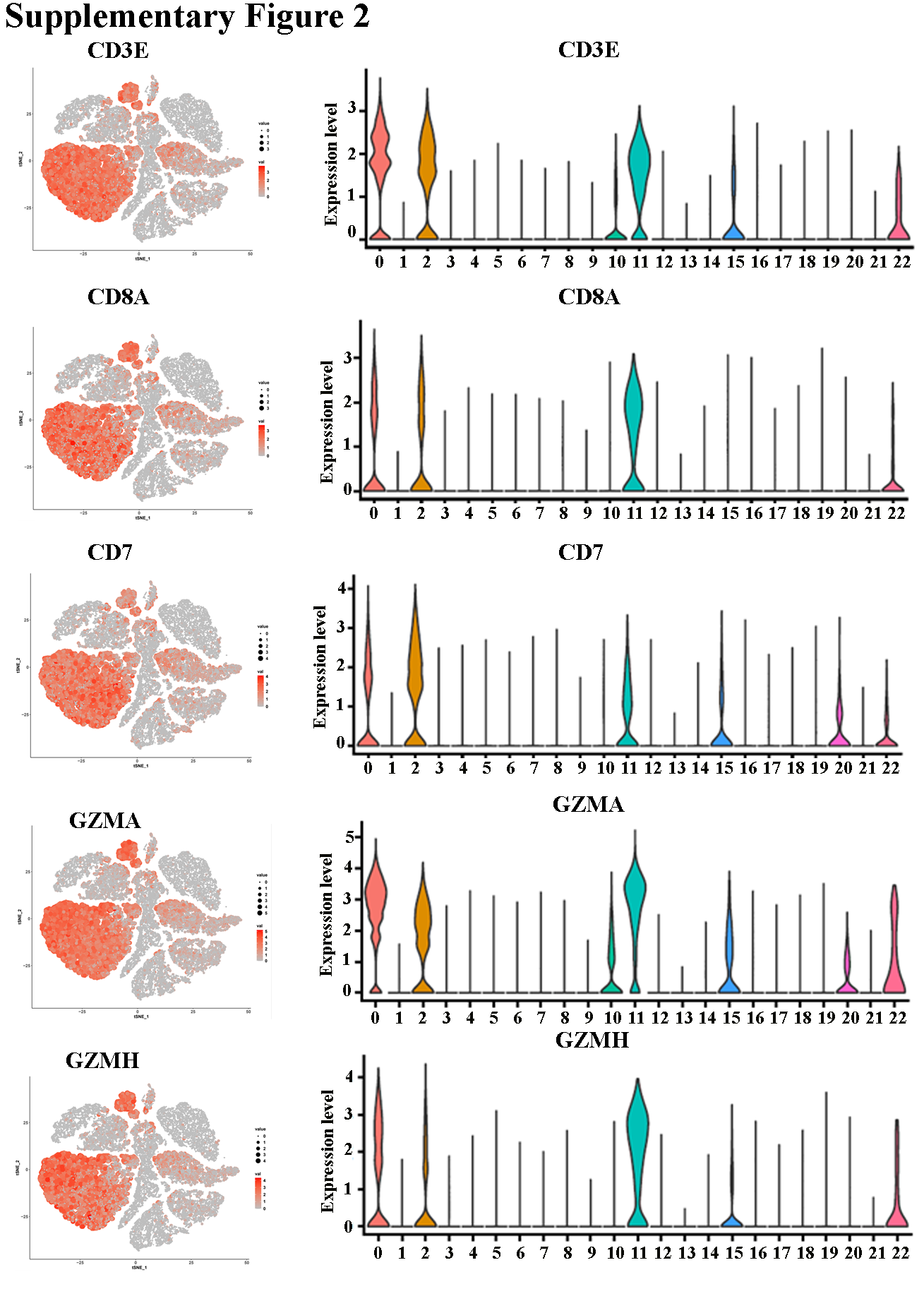

Supplement: Supplementary file 2 [file Image_2.tif]

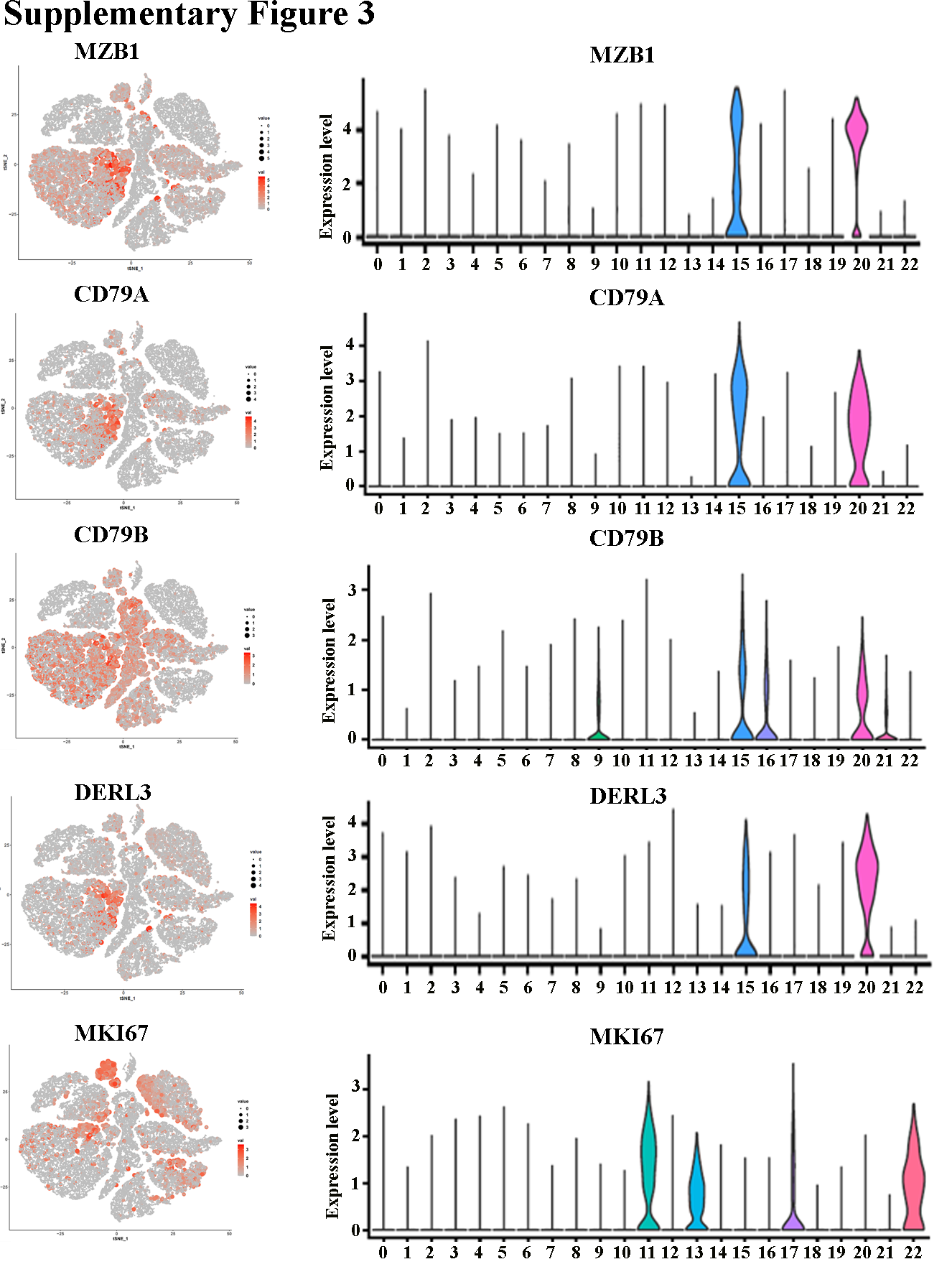

Supplement: Supplementary file 3 [file Image_3.tif]

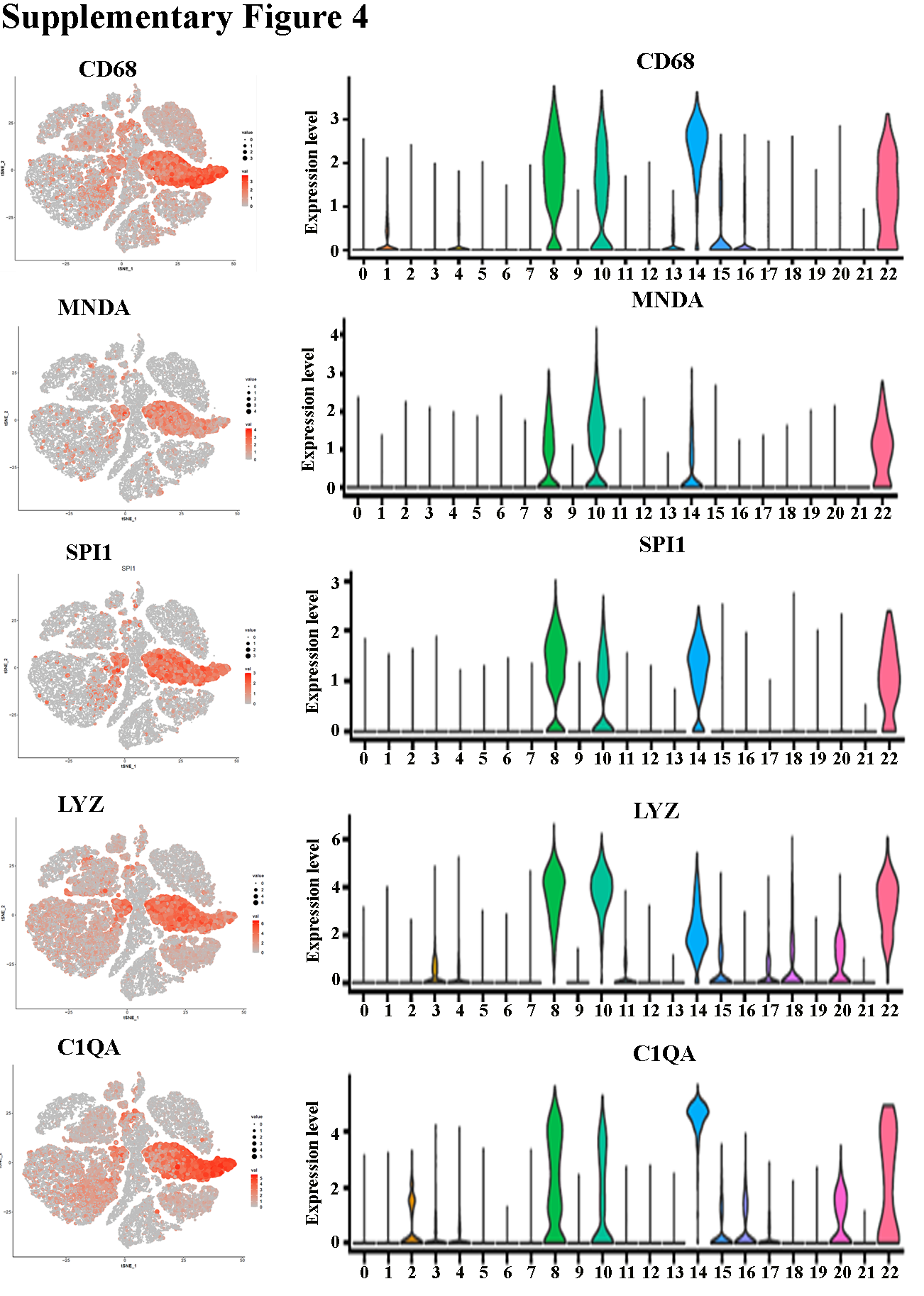

Supplement: Supplementary file 4 [file Image_4.tif]

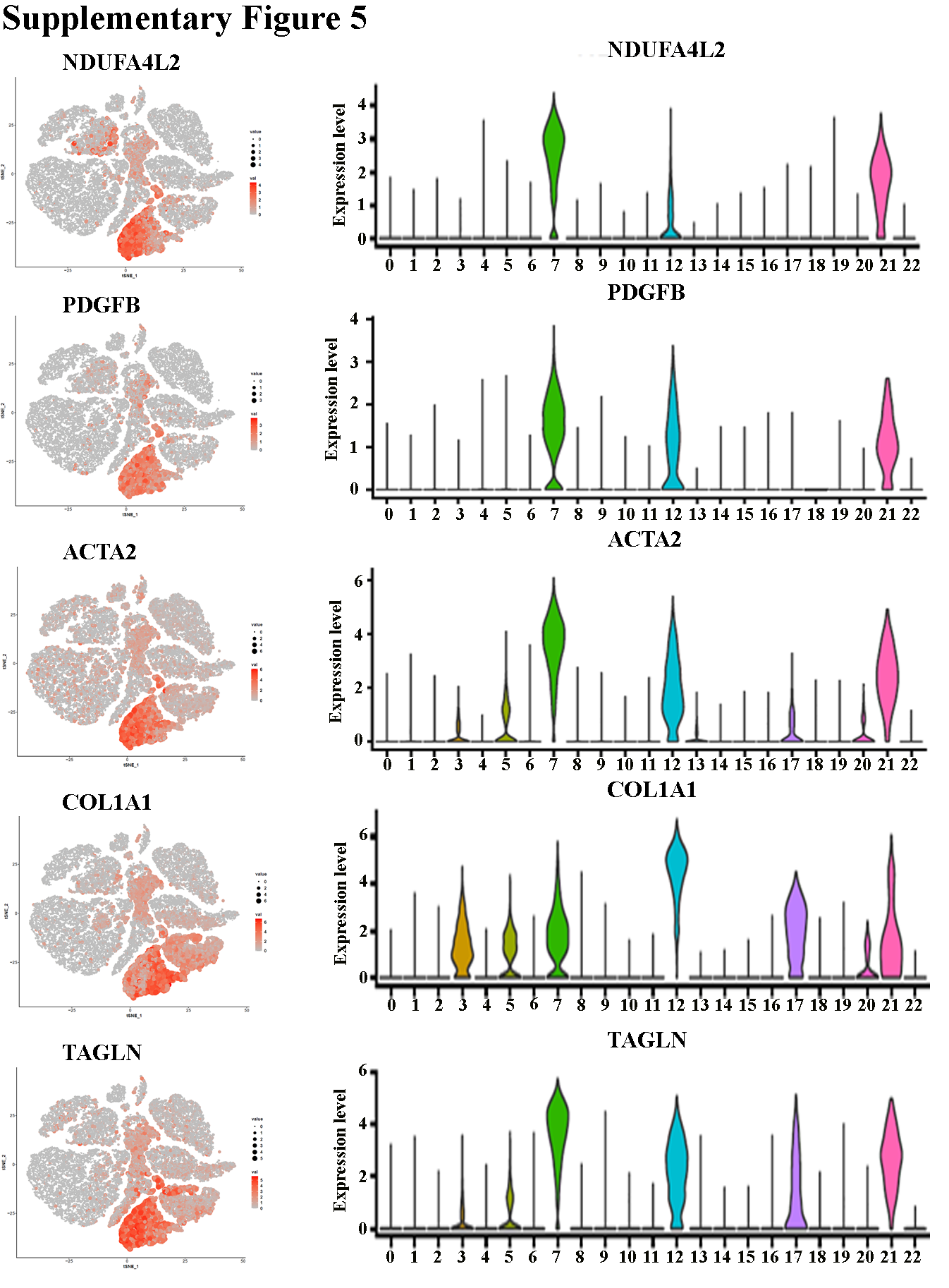

Supplement: Supplementary file 5 [file Image_5.tif]

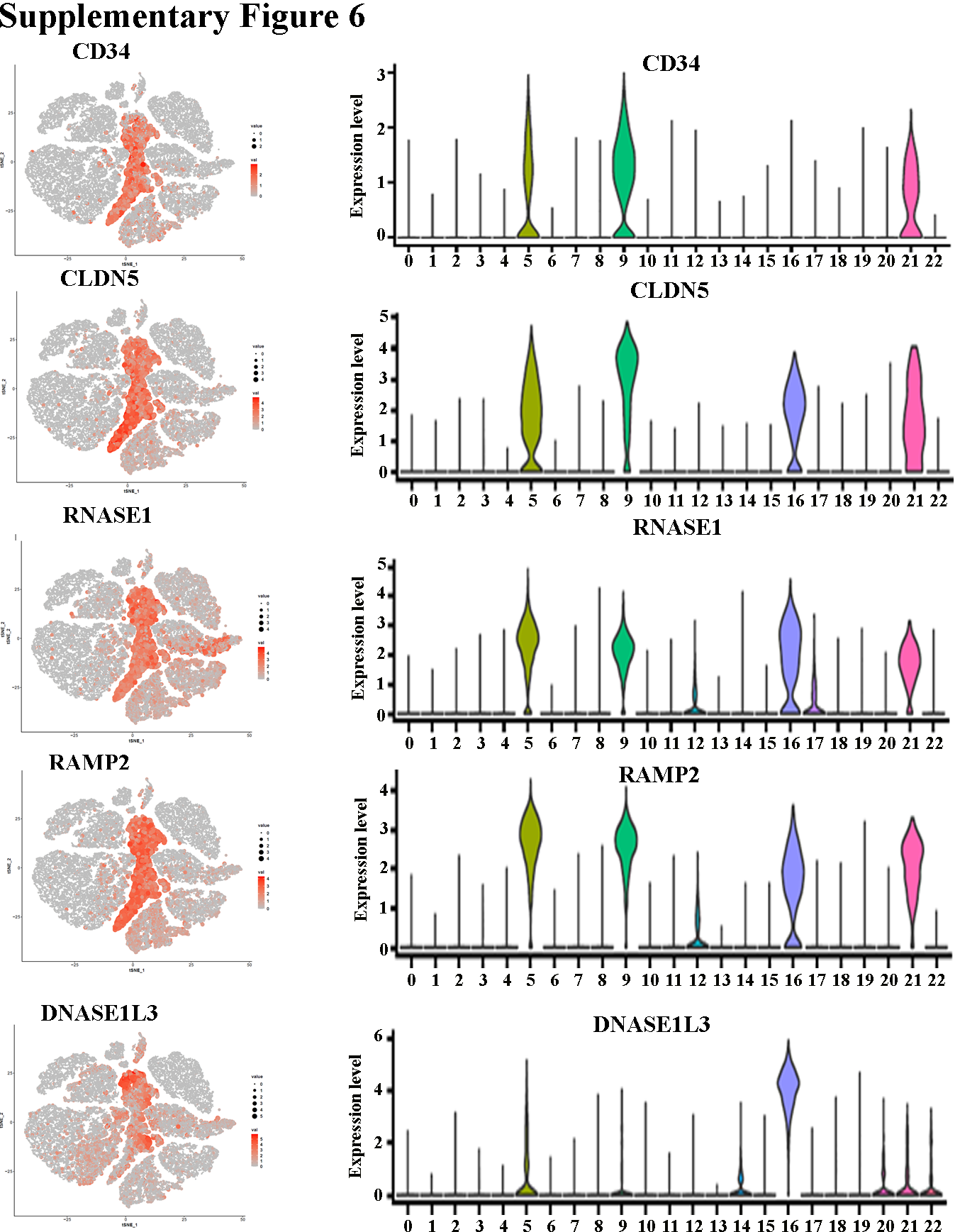

Supplement: Supplementary file 6 [file Image_6.tif]

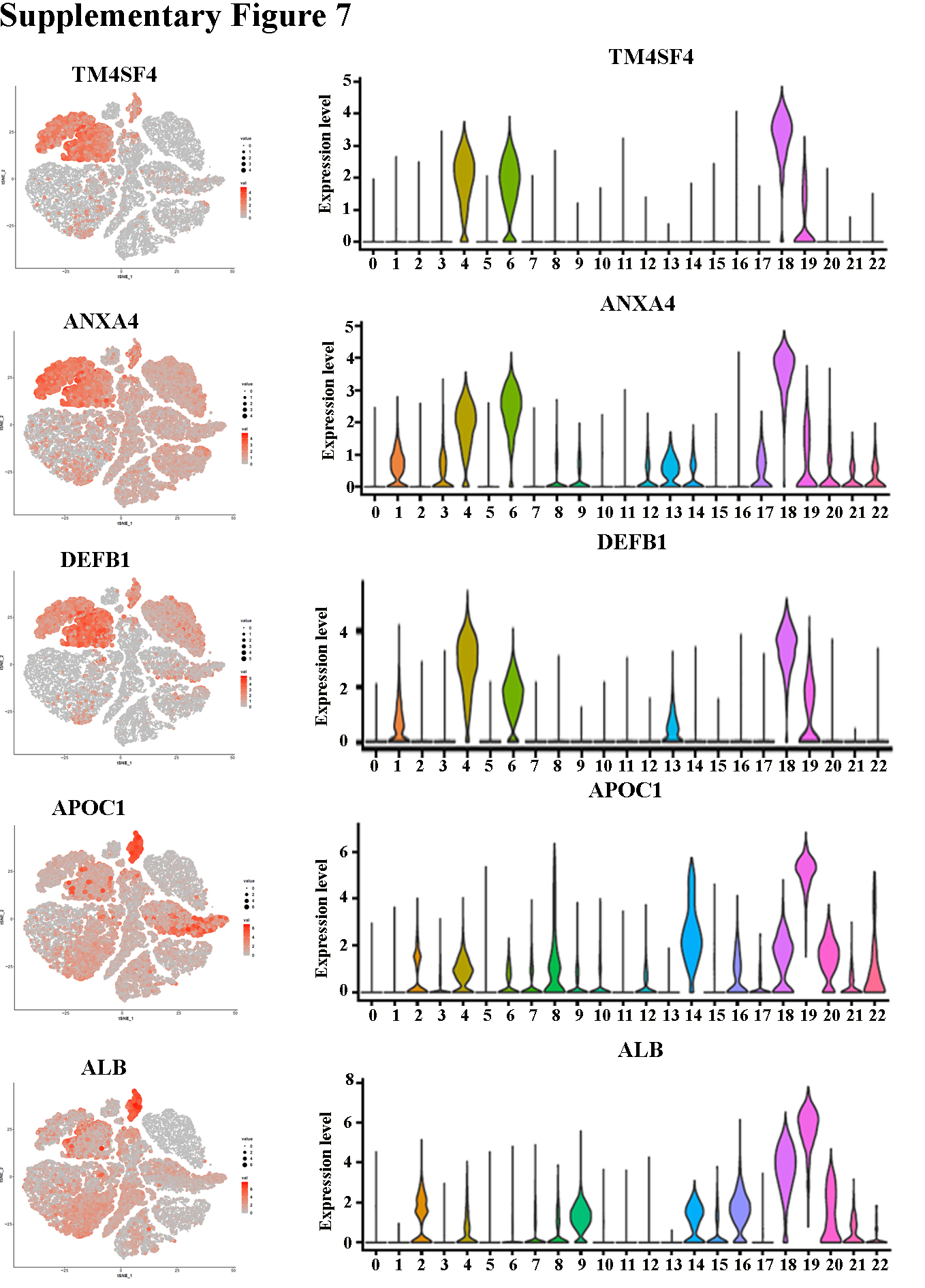

Supplement: Supplementary file 7 [file Image_7.tif]

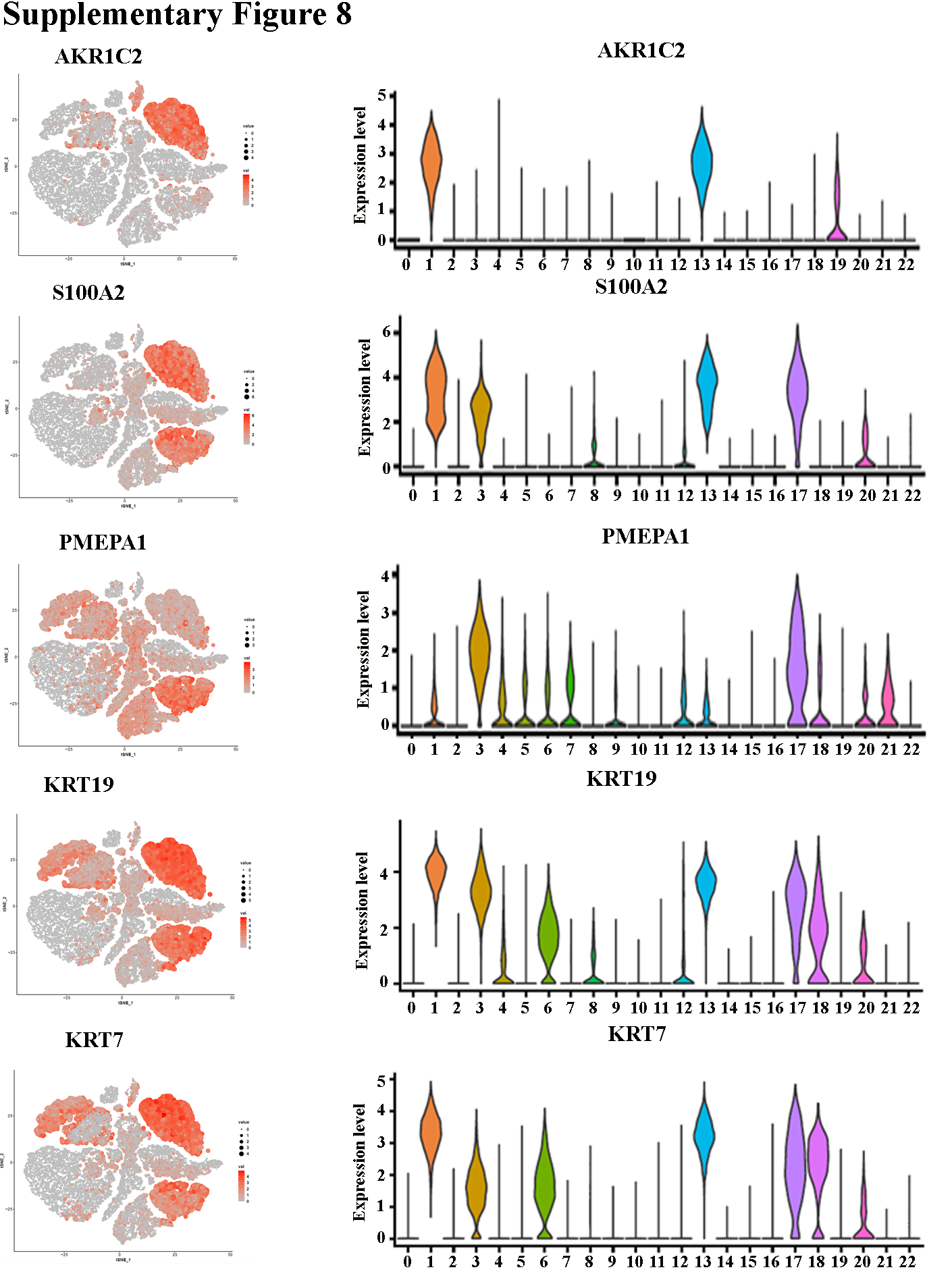

Supplement: Supplementary file 8 [file Image_8.tif]
